# Masking interferes with haptic texture perception from sequential exploratory movements

**DOI:** 10.3758/s13414-021-02253-w

**Published:** 2021-03-11

**Authors:** Knut Drewing, Alexandra Lezkan

**Affiliations:** Institute for Psychology, Justus-Liebig University Giessen, Otto-Behaghel-Str. 10F, Gießen, 35394 Germany

**Keywords:** Haptics, Perception and Action, Texture

## Abstract

Haptic texture perception is based on sensory information sequentially gathered during several lateral movements (“strokes”). In this process, sensory information of earlier strokes must be preserved in a memory system. We investigated whether this system may be a haptic sensory memory. In the first experiment, participants performed three strokes across each of two textures in a frequency discrimination task. Between the strokes over the first texture, participants explored an intermediate area, which presented either a mask (high-energy tactile pattern) or minimal stimulation (low-energy smooth surface). Perceptual precision was significantly lower with the mask compared with a three-strokes control condition without an intermediate area, approaching performance in a one-stroke-control condition. In contrast, precision in the minimal stimulation condition was significantly better than in the one-stroke control condition and similar to the three-strokes control condition. In a second experiment, we varied the number of strokes across the first stimulus (one, three, five, or seven strokes) and either presented no masking or repeated masking after each stroke. Again, masking between the strokes decreased perceptual precision relative to the control conditions without masking. Precision effects of masking over different numbers of strokes were fit by a proven model on haptic serial integration (Lezkan & Drewing, *Attention, Perception, & Psychophysics 80*(1): 177–192, 2018b) that modeled masking by repeated disturbances in the ongoing integration. Taken together, results suggest that masking impedes the processes of haptic information preservation and integration. We conclude that a haptic sensory memory, which is comparable to iconic memory in vision, is used for integrating sequentially gathered sensory information.

## Introduction

Perception refers to the processes of organizing and integrating sensory information in order to give meaning to detected stimuli, while sensation is the process during which sensory information is received and provided to perception (Wolfe et al., 2012). For haptic perception, sensation occurs during sequential exploratory hand movements, and, hence, haptic perception includes the serial integration of sensory information as a fundamental task (Henriques & Soechting, 2005; Klatzky & Lederman, 1999; cf. Gibson, 1966). Typical exploratory movements extend over time and space, gathering repeated information. For example, if we aim to judge the texture of the table in front of us, we will usually perform a lateral finger movement across the table’s surface, and repeat this lateral movement (“stroke”) several times until we reach a decision (Klatzky & Lederman, 1999; Lederman & Klatzky, 1987; Lezkan, Metzger, & Drewing, 2018). Each of the strokes will gather sensory information, which we integrate into a combined percept of the texture (Drewing & Ernst, 2006; Lezkan & Drewing, 2018b; Metzger, Lezkan, & Drewing, 2018). How is such information preserved and integrated over time?

Sensory memory retains comprehensive traces of sense-specific, sensory stimulus information for a very short duration, after stimulus disappearance. A sensory memory, later referred to as iconic memory, was first detected by Sperling (1960) for the visual sense. He briefly flashed letter arrays. If participants were asked to report everything (“whole report”), they only were able to report four to five letters. However, when a cue was presented immediately after the letter array, which indicated that only one specific row needed to be reported (“partial report”), participants were almost perfectly able to report any of the presented rows. The later, however, the cue was presented after the disappearance of the array, the lower was the advantage of the partial report, and it vanished completely with cue delays around 500 ms. These findings imply that all letters are immediately stored in a sensory memory, which, however, decays within around 500 ms (Sperling, 1960). An auditory equivalent of iconic memory is referred to as echoic memory (Neisser, 1967), which has, for example, been demonstrated in an auditory whole–partial report paradigm: If auditory material (spoken letters/digits) was simultaneously presented in three different locations (left ear, right ear, in-between), the subsequent cueing of one location (partial report) resulted in higher percentages of reported material as compared with a whole report (Darwin, Turvey, & Crowder, 1972). Both iconic and echoic memory has been discussed to comprise a more sensory-specific component that lasts shorter (a few 100 ms) and another more abstract component lasting longer (Cowan, 1984; Long, 1980; but cf. Coltheart, 1980).

Iconic and echoic memory have been attributed key roles in integrating serially obtained information to produce a single composite percept (Di Lollo, 1980; Eriksen & Collins, 1968; Sugita, Hidaka, & Teramoto, 2018). For example, iconic memory seems to allow for easy detection of a change in a continuous stream of visual input, while interrupting this stream by a blank screen or an eye blink has yielded the phenomenon of change blindness (Becker, Pashler, & Anstis, 2000). Serial integration is even more prominent in audition, in which typical input signals are sequential in nature. Auditory integration is required, for example, for the categorization of consonants or to preserve speech information required later to understand a sentence or an initially not attended sound (Bendixen & Schröger, 2017). Masking interferes with integration. For example, if in Sperling’s original visual experiment, a random pattern of letter-like visual features or a homogenous light field was presented directly after the to-be-produced letter array, contents of iconic memory were lost faster (Averbach & Sperling, 1961; Gegenfurtner & Sperling, 1993). Masking data have been interpreted in terms of memory erasure, or in terms of interferences resulting from the integration of target and mask into a single composite percept (Eriksen & Hoffman, 1963; Haber, 1970; Kahneman, 1968; Liss, 1968; Turvey, 1973).

Here, we ask whether a sensory memory may play a role for the serial integration of redundant sensory information in active haptic touch. A few studies have suggested a haptic equivalent of iconic and visual memory, indicated by an advantage of partial reports in haptic localization of a set of stimuli (Auvray, Gallace, & Spence, 2011; Bliss, Crane, Mansfield, & Townsend, 1966; Gallace & Spence, 2009; Gallace, Tan, Haggard, & Spence, 2008). Gallace et al. (2008) presented vibrotactile stimulation on multiple body parts in parallel. Participants were asked to either report the total number of stimulations/stimuli positions (whole report) or to judge whether a cued position had been previously stimulated (partial report). Advantages of the partial report decreased with an increasing delay between stimulation and cue within a few seconds. Similarly, Shih, Dubrowski, and Carnahan (2009) found evidence for a short-lived (around 2 s) memorization of object mass used for programming forces in object lifting and concluded from these data on a haptic sensory memory.

A haptic sensory memory would be an appropriate mechanism to preserve and integrate redundant haptic information gathered during the exploration of a stimulus. Sensory memories are involved in information integration in the range of a few seconds, which covers observed durations of the haptic exploration of single stimuli (Lezkan et al., 2018; Lezkan & Drewing, 2018a). One characteristic of sensory memories is that masking interferes with its integration capabilities, erasing or perturbing the content of the sensory memory (Averbach & Sperling, 1961; Gegenfurtner & Sperling, 1993). In the present study, we aimed to study whether haptic sensory memory contributes to the integration of haptic texture information by masking the sensory input during the exploration of texture stimuli. Important evidence for serial information integration during haptic exploration stems from the observation that the precision in discrimination tasks systematically increased with the extension of the exploration—for example, the number of strokes across a texture (=lateral finger movements in a single direction; Hernández-Pérez, Rojas-Hortelano, & Lafuente, 2020; Lezkan & Drewing, 2018b; Metzger et al., 2018). We expect that masking interferes with sensory memory and can diminish the advantage of integrating information from additional strokes. We test that hypothesis in two experiments using an active texture discrimination task in that participants successively stroke several times across each of two textures.

Extended exploration and information integration come along with continuous change. The study of certain aspects of these dynamic processes may call for a dynamic perspective (e.g., Ahissar & Assa, 2016; Beek, Peper, & Daffertshofer, 2002). Here, we approached these issues from a representational perspective—that is, we focused on stable final and intermediate perceptual states and the cognitive mechanisms that underlie their formation. We did so under relatively well-defined conditions (e.g., of exploration). This perspective had been proven fruitful in previous studies (Lezkan & Drewing, 2018a, 2018b; Metzger et al., 2018). It also allowed us to simplify the description of the exploratory movement and information gathering by its discretization into successive stroke units.

## Experiment 1

In the first experiment, participants performed three strokes per texture. Between the strokes across the first texture, we either strongly masked a potential sensory memory by presenting a high-energy tactile pattern, or we presented minimal stimulation through a low-energy smooth surface. High and low energy referred to the intensity of the expected responses of tactile mechanoreceptors to the two patterns. According to what is known on typical responses (e.g., Muniak, Ray, Hsiao, Dammann, & Bensmaia, 2007), we expected firing intensity to be much higher for the high-energy as compared with the low-energy-pattern. In control conditions, no intermediate surface was inserted while three strokes or one stroke is performed. We expected that due to sensory integration, the precision of the percept (assessed by just noticeable differences) is lower in three-strokes control as compared with the one-strokes control condition. Further, we expected that the mask would interfere with sensory memory, and that perceptual precision was reduced in the mask condition as compared with the three-stroke control and the smooth condition. If information from strokes prior to the mask would be completely erased, precision in the masking condition could even have been reduced to that in the one-stroke control condition.

### Methods

#### Participants

Based on the expectation of large effects of masking (Cohen’s *d*_*z*_ = 0.8), the usage of paired *t* tests (for the core analyses of just noticeable differences [JNDs]), a power of 80%, and an alpha level of 5% required sample size was computed for both experiments in advance to be 15 participants for two-tailed testing (Experiment 1) and 12 participants for one-tailed testing (Experiment 2, using G*Power by Faul, Erdfelder, Lang, & Buchner, 2007). In Experiment 1, a total of 16 healthy naïve participants, students from Giessen University, were tested (age range: 18–25 years; 14 females). Data of one participant were excluded because of an outlying bad performance in the task (see Data Analysis section). All participants were right-handed and had two-point discrimination thresholds at the tip of the right index finger of 3 mm or better. Participants took part for course credit and gave written informed consent. The study was approved by the Local Ethics Committee of FB 06 (LEK-FB06) and was conducted in line with the 2013 declaration of Helsinki.

#### Setup and stimuli

In a quiet room, participants sat at a table. They were blindfolded, and their chin was placed on a chin rest to warrant a constant body position. The experimenter sat to the side of the participants; a laptop computer was used to note down the participants’ responses and inform the experimenter about the to-be-presented stimuli. The two stimuli of each trial were placed side-by-side on a small holder, directly in front of the participant around the body midline, and oriented to be explored along a left–right axis. The holder prevented the stimuli from sliding across the table during active exploration.

**Fig. 1:**
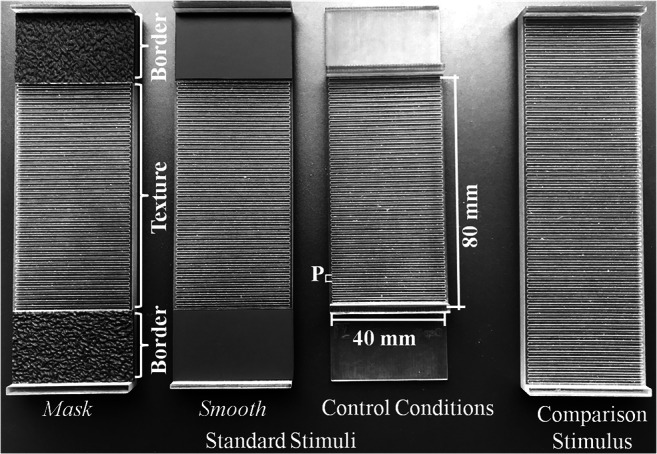
Overview of the standard and comparison stimuli

Haptic grating stimuli were created using the OpenSCAD software and 3D printing (Objet30Pro, StratasysLtd., USA) with model photopolymer material (VeroClear) at a build resolution of 600 × 600 × 1,600 dpi (*x*, *y*, *z* axes). The stimuli (see Fig. 1) were cuboids of 138 mm length (*x*-axis), 42 mm width (*y*-axis) and 6.5 mm height (*z*-axis). Stimuli were subdivided into comparison and standard stimuli (see Fig. 1). Standard stimuli were composed of a textured area, centered at the stimulus’ upper surface (40-mm width and ~80-mm length), and the border area (~26-mm length) on the left and right of it. In experimental conditions, we manipulated the border area by gluing either a plastic shoe sole with a random pattern of surface elements (as the high-energy haptic mask) or contact paper (as the low-energy smooth surface) to it. A barrier, in form of a ridge (3 × 42 × 4 mm), defined the start- and endpoints of strokes. In experimental conditions, the barriers were placed at both ends of the standard stimulus. In control conditions, barriers were placed at both ends of the textured area, which prevented participants from touching the border areas. Comparison stimuli were textured along the full stimulus length (~132 mm) with the barriers at both stimulus ends. The textured area was composed of rectangular ridges and grooves. Grooves and ridges were equally wide; groove depth equaled groove width. Textures were defined by their period P (groove width + ridge width). Standard stimuli had periods of P = 1.69 mm, or P = 1.95 mm, for the comparisons we used stimuli with values between P = 1.35 mm and 2.03 mm or P = 1.61 mm and 2.29 mm, respectively (nine stimuli per series, step size ~0.085 mm).

#### Design and procedure

The experimental design included four within-participants conditions: In two control conditions, participants performed one stroke or three strokes only over the textured area of the control standard stimulus (one-stroke control and three-strokes control condition). In two experimental conditions, participants explored with three strokes the textured and the border area of the standard stimulus (mask and smooth condition). We used the method of constant stimuli combined with a two-interval forced-choice task (2IFC) to assess JNDs for each condition. In each condition, each standard stimulus was paired 10 times with each of the nine comparison stimuli of the respective series—providing 180 trials per condition, which should be an appropriate number to obtain reliable individual JND estimates (cf. O’Regan & Humbert, 1989; Wichmann & Hill, 2001). We used two slightly different values for the standard stimuli rather than one in order to avoid memorization of the standard’s value across trials, which would have invalidated our attempt to study sensory memory. Collapsing data over different standard stimuli could be prone to the problem that due to Weber’s law JNDs for the two stimuli are not exactly the same. However, with the present standard stimuli, such effects should have been quite small and they should have affected all conditions similarly. The experiment was conducted in two sessions. Trials from each condition were presented in separate blocks per session. The order of blocks was counterbalanced between participants by a Latin square design. Within each block the order of trials was randomized.

In each trial, initially one standard and then one comparison stimulus was explored. The standard grating was randomly either presented at the right or the left side. If needed the experimenter placed the participant’s right index finger at the starting position of each stimulus. Participants were instructed to move with a velocity of 14 cm/s during the exploration of the standard stimulus. We enforced the instructed velocity using a metronome: the interval between two beats defined the duration of a single stroke. We did not instruct a specific force in order not to overburden participants. However, it has been shown that in roughness perception participants anyway used relatively constant force within and over trials when exploring a specific texture (Tanaka, Tiest, Kappers, & Sano, 2014). There were no constraints on the exploration of the comparison stimulus. After exploring both stimuli, participants were instructed to move their index finger to a waiting position and to judge which stimulus had had a higher spatial frequency (explained as “number of structural repetitions per spatial segment”). The experimenter noted down the participant’s verbal response in a computer sheet and placed the stimuli in the holder for the upcoming trial.

Overall, session 1 started with the consent form, assessment of spatial discrimination thresholds at the index finger of the dominant hand using a two-point discriminator, instructions, and four practice trials. In each session four blocks of the proper experiment were conducted, one per condition. In total, there were 4 (condition) × 18 (stimulus pairs) × 10 repetitions = 720 trials, which were presented in about 3 hours per session. Both sessions took place within about a single week.

#### Data analysis

**Fig. 2:**
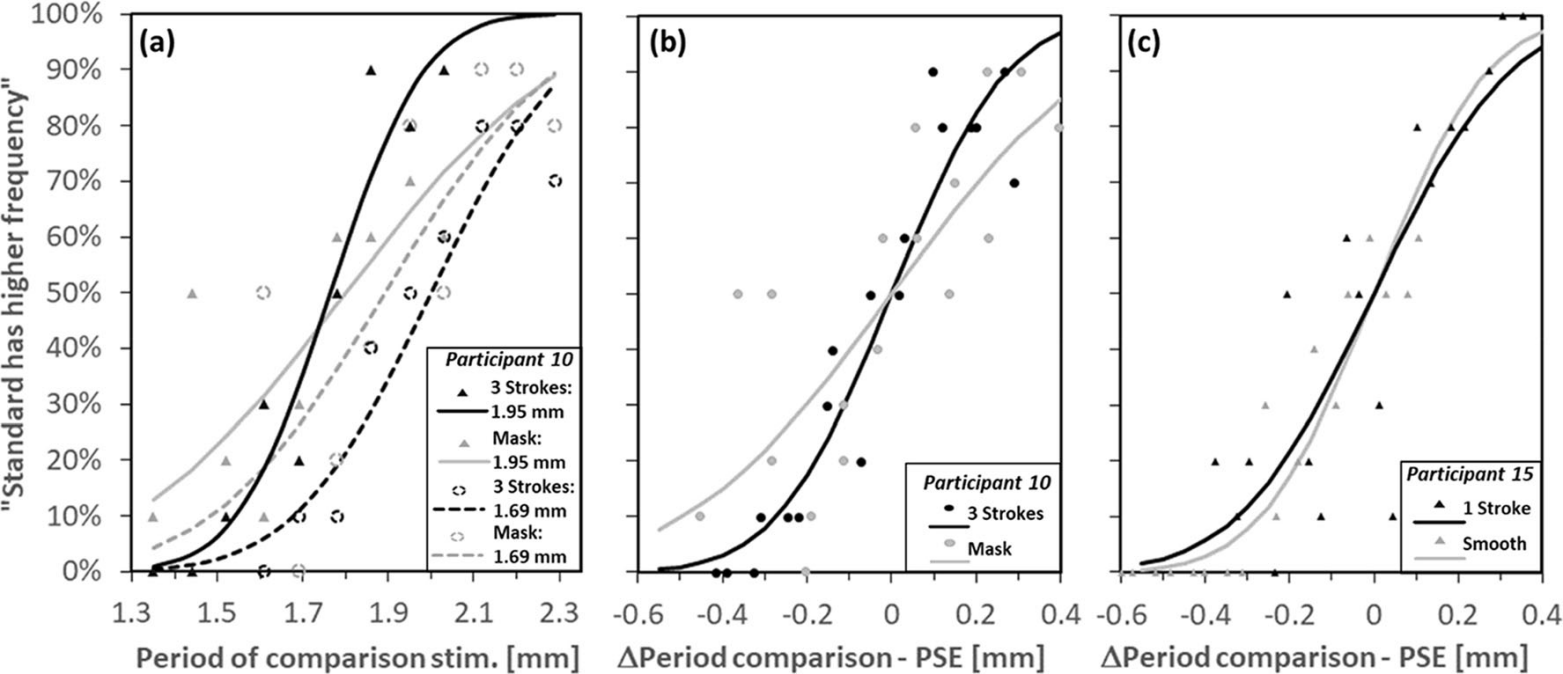
Response data (markers) and psychometric fits by cumulative Gaussians (lines) for exemplary conditions of two participants in Experiment 1: **a** Response frequencies of participant 10 for the masking and the three-strokes control condition as a function of the period of the comparison stimulus separated by the two standard stimuli (periods: 1.69 and 1.95 mm, initial analysis). **b** Response frequencies of the same participant and conditions collapsed over standard stimuli as a function of the period of the comparison stimulus minus PSE. **c** Collapsed frequencies of participant 15 for the smooth and the one-stroke control condition

We fitted individual cumulative Gaussian functions (free parameters μ, σ) to the proportion of trials, in which the comparison was reported to have the higher spatial period (=lower spatial frequency) than the standard as a function of the comparison’s period (psignifit4 toolbox for MATLAB; Schütt, Harmeling, Macke, & Wichmann, 2016). In a first step, we had to align the data from the two standard stimuli per condition. For that purpose, we initially fit individual functions per condition and standard (see Fig. 2a), from which we estimated points of subjective equality (PSE) per standard only (from μ which is the period associated with the 50% proportions). Individual data from the two standards per condition were collapsed by aligning the two data sets at their respective PSEs (i.e., determining the proportion of trials in which the comparison was reported to have the higher spatial period than the standard as a function of the comparison’s period minus PSE; see Fig. 2b–c). Fits to these data were used to assess the JND, which is the difference between the periods associated with 50% and 84% proportions. A lower JND indicates better perceptual precision as compared with a higher JND. Data from participants that showed an extremely bad precision (2.5 standard deviations above average JND) were excluded from analysis (one participant in the present experiment). We checked PSE and JND values per condition on deviation from normality using Kolmogorov–Smirnov tests (Bonferroni corrected for number of tests per type of value). None of these tests reached significance, neither in Experiment 1 nor in Experiment 2. We hence analyzed PSEs and JNDs using analyses of variance (ANOVAs) and planned *t* tests, respectively. We used one-tailed *t* tests for testing directed hypotheses on JNDs, and two-tailed *t* tests otherwise. The *p* values were corrected according to Huynh and Feldt (1976) if required. Effect sizes were given as partial eta square (η_p_^2^) for ANOVAs and Cohen’s *d*_*z*_ for *t* tests.

### Results

First, we submitted PSEs to an ANOVA with the variables standard stimulus (1.69 vs 1.95 mm) and experimental condition (one-stroke control, mask, smooth, three-strokes control; see Table 1). As should be the case, PSEs were higher for the standard with period 1.95 mm as compared with 1.69 mm, *F*(1, 14) = 252.8, *p* < .001, η_p_^2^ = 0.95 (averages: 1.97 vs. 1.77 mm). Unexpectedly, PSE also showed a main effect of experimental condition, *F*(3, 42) = 3.57, *p* = .041, η_p_^2^ = 0.20, and a significant interaction, *F*(3, 42) = 11.44, *p* < .001, η_p_^2^ = 0.45. Pair-wise Bonferroni-corrected post hoc *t* tests between either two experimental conditions separated by standard stimulus revealed that for one standard stimulus (1.95 mm) the PSE in the three-stroke control condition was significantly higher than in the condition with the high energy mask, *t*(14) = 4.81, *p*_*corr*_ = .003. None of these other 12 post hoc comparisons reached significance (overall α = .05).

**Table 1:** Experiment 1, PSE values (in mm, *SEM* in parentheses)

	One stroke	Mask	Smooth	Three strokes
Standard 1.69 mm	1.77 (0.11)	1.74 (0.14)	1.75 (0.18)	1.77 (0.07)
Standard 1.95 mm	2.02 (0.09)	1.87 (0.14)	1.94 (0.15)	2.05 (0.06)

**Fig. 3:**
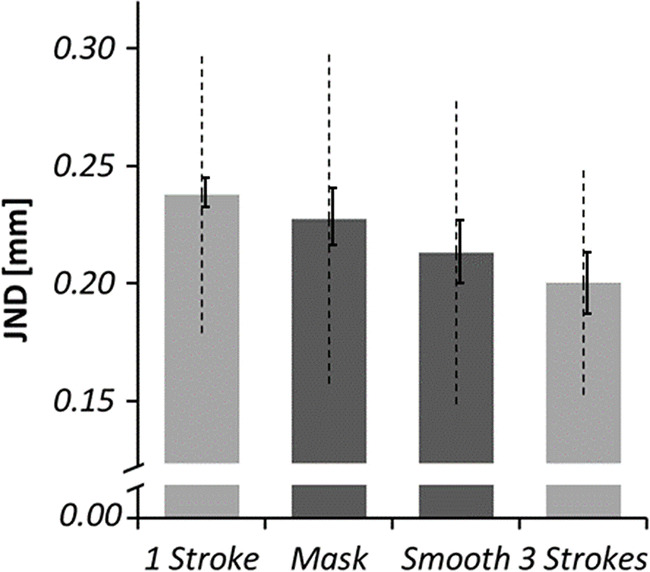
Average JNDs, between-participant standard deviation (dashed), and within-participant standard errors after Morey (2008, solid), Experiment 1. Experimental conditions are depicted in dark gray and control conditions in light gray

Further, we computed planned pair-wise comparisons between the JNDs of different experimental conditions (see Fig. 3). As expected, JNDs obtained while exploring with the high-energy mask were higher than in the three-strokes control condition, *t*(14) = 1.81, *p* = .046 (one-tailed), *d*_*z*_ = 0.47, and not significantly different from the one-stroke control condition, *t*(14) = 0.84, *p* = .418, *d*_*z*_ = 0.21. Also as expected, JNDs gathered with the low-energy surface were lower than in the one-stroke control condition, *t*(14) = 2.38, *p* = .016 (one-tailed), *d*_*z*_ = 0.61, and not significantly different from the three-strokes control condition, *t*(14) = 0.73, *p* = .480, *d*_*z*_ = 0.19. Finally, as expected JNDs were lower in the three-stroke condition as compared with the one-stroke condition, *t*(14) = 3.63, *p* = .001 (one-tailed), *d*_*z*_ = 0.94. Unexpectedly, JNDs in the high-energy mask condition were not significantly higher as compared with the low-energy-mask condition, *t*(14) = 0.97, *p* = .175 (one-tailed), *d*_*z*_ = 0.25.

### Discussion

Taken together, masking between the strokes decreased perceptual precision (larger JNDs) by a significant amount relative to the three-strokes control condition and approached performance in the one-stroke condition. In contrast, the smooth low-energy surface did not significantly reduce precision compared with the three-strokes condition, and performance was better than in the one-stroke control condition, as expected. Overall, the results showed that masking can impede the process of information preservation during haptic exploration, and thus support our hypothesis that haptic sensory memory is used for storing information from different strokes. Although the difference between the mask and the smooth condition was smaller than expected, this may be explained by some residual intermediate low-energy stimulation by the contact paper as compared with the control condition. However, this again might reflect masking and support our hypotheses.

In addition, we found that the same grating period of one of the standards (1.95 mm) was judged to be significantly larger in the three-strokes control condition (2.05 mm) as compared with the high-energy masking condition (1.87 mm). In the General Discussion, we discuss the potential origin of this noticeable effect, which occurred only for one of the two standard stimuli. Here, it is important to note that this effect does not provide an alternative explanation for observed effects on perceptual precision. Given Weber’s law, a higher PSE may come along with a higher JND (lower precision), but in the present three-strokes control conditions, JNDs were lower than in the high-energy masking condition. It might also be noteworthy that PSEs tended to be slightly above the values of the respective standard. Given the standard was always the first stimulus in a trial, this was most likely an order effect probably due to adaptation processes. Importantly, also this effect does not affect our conclusions from JND differences.

Taken together, Experiment 1 showed that masking impedes memory storage during haptic perception. If indeed haptic sensory memory is the basis for the integration of information over time, repeated masking of sensory memory should systematically reduce the perceptual benefits from integration. We tested this hypothesis in Experiment 2.

## Experiment 2

In Experiment 2, we varied the number of strokes across the standard (one, three, five, or seven strokes) and either presented no masking or repeated masking after each stroke. Given that haptic information is integrated over time in order to improve perception (Hernández-Pérez et al., 2020; Klatzky & Lederman, 1999; Lezkan & Drewing, 2018b; Metzger et al., 2018), we expected that without masking, perceptual precision would increase with the number of strokes. With masking, we expected perceptual precision to be overall lower. In addition, if information from the entire exploration is stored and integrated in sensory memory, with longer exploration we expected that this info was masked repeatedly and that the increase of perceptual precision with additional strokes was less pronounced than without masking. If masking would completely erase prior information, masking could even have hindered any precision benefit from integration over strokes.

### Methods

A total of 13 healthy naïve participants, students from Giessen University, were tested (age range: 19–32 years; nine females). Data from two participants were excluded due to outlying performance. All participants were right-handed, and had two-point discrimination thresholds at the tip of the right index finger of 3 mm or better. Participants were paid 8€/h. We used the same setup, all comparison stimuli, and the standard stimuli of the control and the high-energy mask conditions from Experiment 1. The experimental design included two within-participants variables: masking and stroke number. Masking refers to the explored standard stimuli (mask vs. control stimuli), and participants in different stroke number conditions were instructed to explore the standard with one, three, five, or seven strokes. Again we combined the method of constant stimuli with a 2IFC task to assess JNDs, and in each condition, each standard stimulus was paired with each of the nine comparisons 10 times. The experiment was conducted in four sessions. In each session, 45 randomly chosen and randomly ordered trials from each of the eight conditions were presented in a separate block. Across sessions, the order of the eight blocks was counterbalanced for each participant. In total, there were 8 (condition) × 18 (stimulus pairs) × 10 repetitions = 1,440 trials, which were presented in about 2.5–3 hours per session. Otherwise, experimental procedures and data analyses methods were the same as in Experiment1.

### Results

First, we submitted PSEs (see Table 2) to an ANOVA with the variables standard stimulus (1.69 vs 1.95 mm), masking (mask vs. no mask), and stroke number (1, 3, 5, 7). As should be the case PSEs were higher for the standard with period 1.95 mm as compared with 1.69 mm, *F*(1, 10) = 80.20, *p* < .001, η_p_^2^ = .89 (averages: 2.02 vs. 1.83 mm). We also found an unexpected Standard Stimulus × Masking interaction, *F*(1, 10) = 17.84, *p* = .002, η_p_^2^ = .64, indicating that for the standard 1.95 mm, but not for 1.69 mm, masked stimuli had a lower PSE than unmasked ones (1.97 mm vs. 2.06 mm, and 1.84 mm vs. 1.83 mm, respectively). Another unexpected effect was that of stroke number, *F*(3, 30) = 4.73, *p* = .019, η_p_^2^ = .32. However, in pair-wise Bonferroni-corrected post hoc *t* tests (six tests) between either two stroke-number conditions, no single comparison reached significance (overall α = .05). Also, other effects in the ANOVA were not significant: masking, *F*(1, 10) = 0.61, *p* = .453, η_p_^2^ = .06; Standard Stimulus × Stroke Number, *F*(3, 30) = 2.11, *p* = .128, η_p_^2^ = .17; Masking × Stroke Number, *F*(3, 30) = 0.09, *p* = .963, η_p_^2^ = .01; three-way interaction, *F*(3, 30) = 1.89, *p* = .161, η_p_^2^ = .16.

**Table 2:** Experiment 2, PSE values (in mm, *SD* in parentheses)

	Standard stimulus	One stroke	Three strokes	Five strokes	Seven strokes
Mask	1.69 mm	1.83 (0.18)	1.84 (0.13)	1.84 (0.16)	1.85 (0.12)
	1.95 mm	1.90 (0.14)	2.01 (0.12)	2.00 (0.12)	1.99 (0.10)
Control	1.69 mm	1.79 (0.12)	1.84 (0.12)	1.84 (0.08)	1.82 (0.09)
	1.95 mm	2.02 (0.11)	2.07 (0.09)	2.07 (0.08)	2.07 (0.09)

**Fig. 4:**
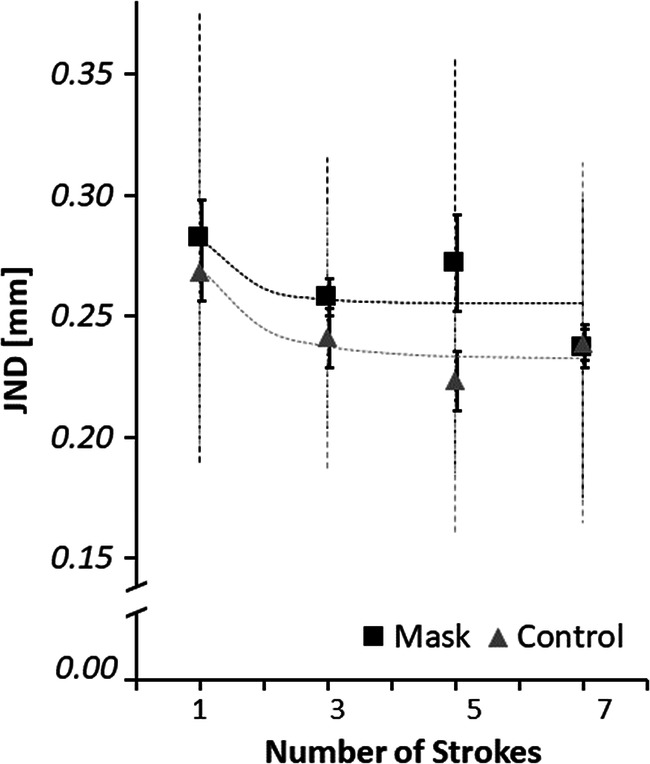
Average JNDs, between-participant standard deviation (dashed) and within-participant standard errors after Morey (2008, solid), Experiment 2, as a function of number of strokes and masking. Dotted lines represent the fit of a serial integration model detailed in the discussion of Experiment 2

Most importantly, we computed planned contrasts between the JNDs (see Fig. 4). As expected, JNDs in masked conditions were higher than in unmasked conditions, *t*(10) = 1.86, *p* = .045 (one-tailed), *d*_*z*_ = 0.59. Also as expected a significant linear contrast of stroke number, *t*(10) = 3.79, *p* = .002 (one-tailed), *d*_*z*_ = 1.20, confirmed that JNDs decreased with an increasing number of strokes. Unexpectedly, the decrease was not significantly higher in the no-mask as compared with the masked condition as was shown by the linear contrast of the Stroke Number × Masking interaction, *t*(10) = 0.04, *p* = .48 (one-tailed), *d*_*z*_ = 0.01.

### Discussion

Again, masking between the strokes decreased perceptual precision by a significant amount relative to the control conditions without masking. This corroborated the view that masking impedes the process of haptic information preservation and that haptic sensory memory is used for storing information (cf. Averbach & Sperling, 1961). The number of strokes had no effect on the magnitude of the masking effect. At first glance, this seems not to fit with the view that redundant stimulus information from the entire exploration is integrated in sensory memory, because then masking effects should have increased with repeated masking. However, masking effects in the present experiment were overall not as large as expected, and any modification of masking effects by stroke number/repetition would even have been smaller. That is, while larger effects of masking would have allowed us to clearly distinguish whether benefits from additional strokes are smaller with versus without masking or not, and thus to directly support or reject our hypothesis, this was difficult with the present small masking effects.

Importantly, the present data can well be modeled by using an existing model on serial haptic integration (Lezkan & Drewing, 2018b) extended by repeated masking. The model assumes that texture estimates ***S***_**1**_^(***j***)^ from each stroke ***j=1…n***_***1***_ across the first stimulus (*1*) in a trial are serially integrated into an overall texture representation $\hat{{\boldsymbol{S}}_{\mathbf{1}}}={\hat{{\boldsymbol{S}}_{\mathbf{1}}}}^{\left(\boldsymbol{n}\mathbf{1}\right)}$. Then, during exploration of the second stimulus (2) in the trial stroke-specific difference scores ***D***^(***i***)^ are computed between the integrated texture representation from the first stimulus $\hat{{\boldsymbol{S}}_{\mathbf{1}}}$ and each stroke-specific estimate from the second stimulus ***S***_**2**_^(***i***)^ (***i=1…n***_***2***_**,** ${\boldsymbol{D}}^{\left(\boldsymbol{i}\right)}=\hat{{\boldsymbol{S}}_{\mathbf{1}}}-{{\boldsymbol{S}}_{\mathbf{2}}}^{\left(\boldsymbol{i}\right)}\Big)$**,** which are serially integrated into an overall estimate of stimulus difference $\hat{\boldsymbol{D}}={\hat{\boldsymbol{D}}}^{\left(\boldsymbol{n}\mathbf{2}\right)}$. Further, it is assumed that the representation from the first stimulus decays during the exploration of the second stimulus. The successive stroke-wise built-up of the first stimulus representation ${\hat{{\boldsymbol{S}}_{\mathbf{1}}}}^{\left(\boldsymbol{n}\mathbf{1}\right)}$, and the difference ${\hat{\boldsymbol{D}}}^{\left(\boldsymbol{n}\mathbf{2}\right)}$are modeled by Kalman filters:

1 $$ {\hat{{\boldsymbol{S}}_{\mathbf{1}}}}^{\left(\boldsymbol{j}\right)}=\hat{{\boldsymbol{S}}_{\mathbf{1}}}{\prime}^{\left(\boldsymbol{j}\right)}+{{\boldsymbol{k}}_{\boldsymbol{S}}}^{\left(\boldsymbol{j}\right)}\left({{\boldsymbol{S}}_{\mathbf{1}}}^{\left(\boldsymbol{j}\right)}-\hat{\ {\boldsymbol{S}}_{\mathbf{1}}}{\prime}^{\left(\boldsymbol{j}\right)}\right) $$

2 $$ {\hat{\boldsymbol{D}}}^{\left(\boldsymbol{i}\right)}=\hat{\boldsymbol{D}}{\prime}^{\left(\boldsymbol{i}\right)}+{{\boldsymbol{k}}_{\boldsymbol{D}}}^{\left(\boldsymbol{i}\right)}\left({\boldsymbol{D}}^{\left(\boldsymbol{i}\right)}-\hat{\ \boldsymbol{D}}{\prime}^{\left(\boldsymbol{i}\right)}\right). $$

The filters start with an initial first representation of stimulus/difference ${\hat{{\boldsymbol{S}}_{\mathbf{1}}}}^{\left(\mathbf{1}\right)}$**,** ${\hat{\boldsymbol{D}}}^{\left(\mathbf{1}\right)}$ based on the information from the respective first strokes (***S***_**1**_^(**1**)^***,D***^(**1**)^). This first representation becomes the prior information for the second stroke $\hat{{\boldsymbol{S}}_{\mathbf{1}}}{\prime}^{\left(\mathbf{2}\right)}={\hat{{\boldsymbol{S}}_{\mathbf{1}}}}^{\left(\mathbf{1}\right)}$, $\hat{\boldsymbol{D}}{\prime}^{\left(\mathbf{2}\right)}={\hat{\boldsymbol{D}}}^{\left(\mathbf{1}\right)}$**,** which is optimally integrated with the novel information ***S***_**1**_^(**2**)^***, D***^(**2**)^ obtained from the second stroke. This process is repeated for each stroke until the final strokes ***n***_***1***_***, n***_***2***_. That the integration is optimal is warranted by the definition of the Kalman gains ***k***_***S***_^(***j***)^ and ***k***_***D***_^(***i***)^, which weigh prior and novel information according to their inverse variances (=reliability; for details see Lezkan & Drewing, 2018b). Further, the model assumes that the variance of each one-stroke based estimate is the same, and the decay of the first-stimulus representation during exploration of the second stimulus is modeled by a stroke-dependent increase of its variance ${\boldsymbol{\sigma}}_{\hat{{\boldsymbol{S}}_{\mathbf{1}}}}^{\mathbf{2}\ \left(\boldsymbol{i}\right)}={\boldsymbol{i}}^{\mathbf{0.442}}{\boldsymbol{\sigma}}_{\hat{{\boldsymbol{S}}_{\mathbf{1}}}}^{\mathbf{2}}$**,** (model and number 0.442 from Murray, Ward, & Hockley, 1975). In Lezkan and Drewing (2018b), the model well predicted the stroke-specific weighting of estimates in a 2IFC texture discrimination task (computed from the Kalman gains).

Albeit not considered in detail in Lezkan and Drewing (2018b), some process noise ${\boldsymbol{\sigma}}_{\boldsymbol{w}}^{\mathbf{2}}$can be assumed to add in each step of the successive integration during the exploration of the first and second stimulus (see Equations 1–2), and used to model repeated masking. Then, with ***w~N(0,*** ${\boldsymbol{\sigma}}_{\boldsymbol{w}}^{\mathbf{2}}\Big)$***:***

3 $$ \hat{{\boldsymbol{S}}_{\mathbf{1}}}{\prime}^{\left(\boldsymbol{j}\right)}={\hat{{\boldsymbol{S}}_{\mathbf{1}}}}^{\left(\boldsymbol{j}-\mathbf{1}\right)}+\boldsymbol{w} $$

4 $$ \hat{\boldsymbol{D}}{\prime}^{\left(\boldsymbol{i}\right)}={\hat{\boldsymbol{D}}}^{\left(\boldsymbol{i}-\mathbf{1}\right)}+\boldsymbol{w}. $$

We used this extended model here to predict JNDs (by ${\boldsymbol{\sigma}}_{\hat{\boldsymbol{D}}}$**,** i.e., the square root of the variance of the final difference estimate as computed from successive integration), given the following: The first stimulus was always the standard stimulus, which varied in the number of applied strokes and the amount of masking after each stroke. The second stimulus was the comparison, for which we assume that it was explored by a relatively constant behavior across conditions. Based on observations made by Lezkan and Drewing (2018a) during the free exploration of similar gratings in a 2IFC task (Experiment 2), we assumed that participants made four strokes on the comparison. The model had three free parameters: the variance of a single stroke-specific estimate, process noise in the masking condition, and process noise in the no-masking condition.

We fit the present average JNDs to the model using least-squares fit methods. The fit explains *R*^2^ = 74% of variance. The variance of a stroke-specific estimate was fit as 0.075 mm^2^, which is highly plausible in that it is similar to a value of 0.099 mm^2^ observed in Lezkan and Drewing (2018a, Experiment 1), with one stroke per stimulus and just slightly different gratings. Process noise in the control condition was fit as 0.012 mm^2^ and with masking as 0.031 mm^2^, which is also plausible: Process noise was much higher in the masking condition and can thus well represent the effects of repeated masking. The small process noise in the control condition might be led back to other processes or to small masking effects at the stimulus’ edges where participants reverted their stroking direction.

Overall, the present data did not allow to directly support the assumption that serial integration is repeatedly disturbed by repeated masking. But they are consistent with this assumption: The data were fit by an extension of an established model on serial integration in that masking is modeled by repeated process noise.

Of course, one may think of other theories to analyze the differences between conditions. For example, masking conditions may be considered to have provided more contextual interference than nonmasking conditions, and theoretical models of contextual interference have suggested that low interference fosters automatic rather than controlled processing (Shea & Zimny, 1988), and promotes retroactive inhibition of similar material (Shea & Graf, 1994). Indeed, automatic processing has been occasionally reported to have better performance in a highly automatized task like haptic perception (cf. Zoeller, Lezkan, Paulun, Fleming, & Drewing, 2019), which we have observed in the nonmasking as compared with the masking conditions. However, retroactive inhibition is hardly a mechanism that is effective in repeated stroking: data showed to the contrary that in such tasks later information is used less frequently than earlier information (e.g., Lezkan & Drewing, 2018a), rendering this alternative interpretation unlikely.

General Discussion

In two experiments, we demonstrated that masking during the exploration of a grating reduced the precision of grating perception. That is, masking impeded the process of information preservation during haptic exploration, supporting our hypothesis that haptic sensory memory is used for storing information from different strokes (Averbach & Sperling, 1961; Gegenfurtner & Sperling, 1993). For vision and audition, iconic and echoic memory have been assumed to have key functions in integrating serially obtained information into a composite percept (Eriksen & Collins, 1968; Sugita et al., 2018). Findings from our Experiment 2 are consistent with extending this notion to haptic sensory memory: Precision effects of repeated masking during serial integration were fit by a proven model that considered the masking by repeated disturbances in an ongoing integration process. Hence, we suggested that, similar to other senses, serial information integration in haptic perception is based on a sensory memory.

The notion that sensory memories are responsible for serial integration implies that masking effects result from the integration of target and mask into a composite percept rather than from memory erasure (Haber, 1970; Liss, 1968; Turvey, 1973)—just as specific stimulus perturbations have affected the percept in serial haptic integration: When, during repeated exploration, movements across a single stimulus (e.g., strokes across texture), the judged stimulus property (e.g., texture’s spatial frequency) was altered for a single exploration movement, as compared with the other movements, the entire percept of the property was slightly shifted toward that alteration (Lezkan & Drewing, 2018b; Metzger et al., 2018). Similar integration effects may underlie the effects of the mask on the point of subjective equality observed in the present study: In both Experiments 1 and 2, masked standard stimuli were judged as having a slightly lower spatial period than corresponding control stimuli without the mask—at least for the standard stimulus with the higher spatial period. Probably, this was because the mask also had a textural pattern with relatively high spatial frequency content, which—when being integrated—reduced the perceived spatial period. The effect might have been particularly pronounced for the higher spatial period stimuli because, here, the discrepancy between stimulus and mask was more pronounced than for the lower spatial period stimuli. Thus, effects of the mask on perceived texture also fit the view that haptic sensory memory underlies serial integration, and that masking reflects interferences resulting from the integration of target and mask into a single composite percept rather than memory erasure (cf. Eriksen & Hoffman, 1963; Kahneman, 1968; Turvey, 1973).

It should be noted that the very idea of a sensory memory itself has sometimes been controversially discussed, in particular, if sensory memory has been considered as a delimitable mental entity. For example, from a direct perception perspective, it is the temporally extended sensory array that provides the basis for perceiving invariants in the world, and an extra sensory memory concept is not necessary (Michaels & Carello, 1981). In line with such a view, there is evidence that different memory-related processes are distributed across different specialized processing areas, building a continuum rather than separate entities (D’Esposito & Postle, 2015). Our data could also be considered from such a view: Also, in a specialized sensory area that is responsible for extended information arrays, more extensive exploration would have improved perception up to a certain level, and interference would have occurred if other objects (here the mask) intervened in the sensory array. If interpreting “sensory memory” and “serial integration” as functions of specialized sensory areas that are responsible for extended information arrays (and our model as being descriptive rather than essentialist), we think that our main theoretical conclusions would not contrast with the above views.

Across which exploration duration can redundant information be integrated? Previous studies have suggested that haptic sensory memories decrease only over a few seconds (e.g., Gallace et al., 2008; Shih et al., 2009). In contrast, some studies showed that perceptual precision did not further benefit from integration when extending the exploration beyond about a second (Drewing, Lezkan, & Ludwig, 2011; Hernández-Pérez et al., 2020; but cf. Klatzky & Lederman, 1999), and this was taken as evidence for a temporal limit of integration around this interval. However, a lack of significant further benefit from extra information does not necessarily mean that integration has reached its temporal limit. Because extra information will cause the less benefit the more information has already been gathered, at some later point in the exploration the added benefit is just hard to be detected experimentally. Even more likely, when only small parts of the information stored in sensory memory get lost over time, at some point the benefit from new information could be outweighed by this loss. Indeed, when we modeled the data from our Experiment 2, we observed a small process noise also in the no-masking condition, which indicated a small permanent loss of information. In addition, model predictions in Fig. 4 (dotted lines) suggested that precision benefits leveled out with longer exploration and that this occurred earlier when the process noise was larger (i.e., for the masked as compared with the unmasked condition). Thus, even when perceptual precision does not benefit any more from new information, it might still be integrated while losing some (not all) older information. In order to find out across which temporal interval haptic sensory memory integrates information, perturbation paradigms could be a promising future approach: They can test in which temporal interval limited alterations of a stimulus property influence the perception of that property.

Another open question is how the perceptual system recognizes/determines when the exploration of one stimulus ends and that of another one begins (i.e., how the perceptual system determines which input information is integrated in sensory memory and when information should be attributed to the next/another object). Related to this question is the even more difficult case of single real-life objects that have multiple textures, such as, for example, the sole and the vamp of a shoe. Usually, we can distinguish these textures and, at the same time, attribute them to the same object. Regarding the present task, we can, for example, ask why masks were integrated into the object’s perception, but information from the two gratings could be kept separate. The integration of masks fits with the conceptualization of sensory memories as an early store receiving the continuous stream of incoming sensory information. Consistent with this concept, sensory memories may process only temporally continuous streams of information, and disturbing information in-between, such as masks, cannot be filtered out. However, it is obviously possible to keep different temporally successive streams apart (e.g., the information from two different stimuli). In the case of a multitextured object, as the shoe, exploration procedures might make a difference: If exploration keeps the different textures temporally and spatially apart, they could well be distinguished, but with other, disadvantageous exploration patterns confusion might also occur in such objects. Previous studies from multisensory integration showed that two (more or less simultaneous) events were perceived as belonging to the same underlying object if they were close in spatial location, time, properties, and structure. In contrast, larger discrepancies tended to lead to the conclusion of two independent causes for the events (Hairston et al., 2003; Kayser & Shams, 2015; Wallace et al., 2004; Warren & Cleaves, 1971). Such inference can be performed continually and effortlessly in multisensory perception and has been modeled by Bayesian inference (Cao, Summerfield, Park, Giordano, & Kayser, 2019; Körding et al., 2007). Similarly, two successive streams of information in haptic sensory memory might be distinguished by systematic discrepancies in perceptual properties and change of location; in the present task, for example, when sensory information on the grating period changes, or the hand moves to another stimulus. Yet Lederman and Klatzky (1987) have emphasized the crucial role for motor enhancement in haptic processing. However, given the observed effects of masks during serial integration, a change in stimulus property alone does not seem to automatically yield to a partition of an ongoing information stream. Stronger cues or intervening higher-level processes may be required. However, these speculations remain to be tested in future experimentation.

Taken together, the present study provided evidence that a haptic sensory memory plays a crucial role for integrating serially gathered stimulus information in haptic perception. The detailed properties of such haptic sensory memory, how it interacts with other subsystems that serve perception, and whether it indeed can be equated with haptic sensory memories evidenced in earlier studies (Auvray et al., 2011; Bliss et al., 1966; Gallace et al., 2008; Gallace & Spence, 2009) provide core question for future research.

#### Acknowledgments and open practices statement

Supported by Deutsche Forschungsgemeinschaft (SFB/TRR135/1-2, A05, Project Number 222641018). Data of individual observers from all experiments presented here will be available at zenodo.org, doi:10.5281/zenodo.3907325, starting from publication of the paper.
